# Supplementary material for: Urban Surface Ozone Concentration in Mainland China during 2015–2020: Spatial Clustering and Temporal Dynamics
Source: Int J Environ Res Public Health. 2023 Feb 21;20(5):3810. doi: 10.3390/ijerph20053810 (PMC10001023; doi:10.3390/ijerph20053810)
Supplement: Supplementary file 1 [file ijerph-20-03810-s001.zip › ijerph-2146763-supplementary.pdf]

*Supplementary information for*

# Urban Surface Ozone Concentration in Mainland China during 2015–2020: Spatial Clustering and Temporal Dynamics

**Youru Yao [1,†], Kang Ma [1,†], Cheng He [2,*], Yong Zhang [3], Yuesheng Lin [1], Fengman Fang [1], Shiyin Li [4] and Huan He [4,*]**

1 Key Laboratory of Earth Surface Processes and Regional Response in the Yangtze-Huaihe River Basin, School of Geography and Tourism, Anhui Normal University, Wuhu 241002, China
2 Helmholtz Zentrum München–German Research Center for Environmental Health (GmbH), Institute of Epidemiology, 85764 Neuherberg, Germany
3 Department of Geological Sciences, University of Alabama, Tuscaloosa, AL 35487, USA
4 School of Environment, Nanjing Normal University, Nanjing 210023, China
* Correspondence: cheng.he@helmholtz-muenchen.de (C.H.); huanhe@njnu.edu.cn (H.H.)
† These authors contributed equally to this work.

## Summary

Number of figures:6; Number of tables:5.

Table S1. Data Types and Sources

| Type | Data | Data description | Year | Source |
|---|---|---|---|---|
| Ground-level air pollutant | $PM_{2.5}$、$PM_{10}$、$NO_2$、$SO_2$、CO、O3 | Hourly-average Observations | 2015-2020 | http://106.37.208.233:20036/ |
| Land cover-related | Digital elevation model (DEM) | Spatial resolution (1 km) | 2018 | http://www.resdc.cn/ |
| | Normalized difference vegetation index (NDVI) | Spatial resolution (1 km) | 2018 | http://www.resdc.cn/ |
| Meteorological elements | Basic Meteorological Data | Precipitation; pressure; wind speed; temperature; relative humidity; sunshine hours | 2018 | http://data.cma.cn |

Table S2. Statistics of the ground-level $O_3$ concentration in different cities/countries

| Regions | Types | Year | $O_3$ concentration ($\mu g/m^3$) | References |
|---|---|---|---|---|
| Russian Territory | Urban stations | 2020 first half | 40 | (Andreev et al. 2020) |
| Iran | Control stations | 2015 | 2-126 | (Ezimand and Kakroodi 2019) |
| Pune, India | Urban stations | 2017 | 10-92 | (Anand et al. 2020) |
| Silesia Region, Poland | Urban stations | 2015 | Max 160 | (Kobza et al. 2021) |
| Hamburg, German | Urban stations | 2000-2017 | Mean 56.3, Max 192.1 | (Krug et al. 2020) |
| Leipzig, German | Urban stations | 2000-2017 | Mean 65.3, Max 198.3 | (Krug et al. 2020) |
| France | Urban sites | 1999-2012 | 98th percentile 46.2 | (Sicard et al. 2016) |
| China | Urban stations | 2018 | 25-230 | This study |

Table S3. Standard deviation elliptic parameters of urban $O_3$ in mainland China from 2015 to 2020

| Year | Xcoord | Ycoord | Long axis/km | Short axis/km | Rotation | Area/km² | North-South distances/km | East-West distances/km |
|---|---|---|---|---|---|---|---|---|
| 2015 | 111.973288 | 33.36638 | 2727.82 | 1841.10 | 79.965774 | 3944147.97 | 0 | 0 |
| 2016 | 111.964956 | 33.364238 | 2697.48 | 1828.38 | 81.429260 | 3873060.80 | -30.34 | -12.72 |
| 2017 | 112.005505 | 33.377605 | 2671.06 | 1828.28 | 81.919845 | 3835207.67 | -26.42 | -0.10 |
| 2018 | 112.086342 | 33.408489 | 2682.62 | 1826.60 | 80.320602 | 3848261.55 | 11.56 | -1.68 |
| 2019 | 111.961225 | 33.169184 | 2660.72 | 1854.44 | 81.130302 | 3875054.93 | -21.90 | 27.84 |
| 2020 | 111.936832 | 33.243016 | 2691.02 | 1855.62 | 81.043078 | 3921629.37 | 30.30 | 1.18 |
| | | | | | | | -36.80 | 14.52 |

Table S4. Fitting parameters of the OLS, GWR and MGWR models

| Model indicators | OLS | GWR | MGWR |
|---|---|---|---|
| Residual sum of squares | 216.299 | 125.966 | 124.541 |
| Log-likelihood | -397.130 | -308.464 | -306.597 |
| AICc | 821.420 | 732.506 | 712.531 |
| $R^2$ | 0.341 | 0.616 | 0.620 |
| Adj. $R^2$ | 0.318 | 0.550 | 0.564 |
| Bandwidth | None | 170 | 326 (Intercept)<br>314 ($NO_2$)<br>150 ($SO_2$)<br>56 ($PM_{2.5}$)<br>298 (DEM)<br>326 (Air pressure)<br>326 (Wind speed)<br>326 (Temperature)<br>217 (Relative humidity)<br>318 (Sunshine hours)<br>108 (Precipitation)<br>132 (NDVI) |

Table S5. Fitting parameters for the interaction of multi-factors on $O_3$ concentration variation based on multi-factor generalized additive model (GAM)

| Factors | edf Ref | df | F-Value | *P*-Value | R-sq.(adj) | Deviance explained(%) | n |
|---|---|---|---|---|---|---|---|
| $NO_2$ | 8.745 | 8.963 | 340.8 | 2e-16*** | 0.0535 | 5.37 | 122739 |
| $SO_2$ | 8.864 | 8.990 | 355.4 | 2e-16*** | | | |
| $NO_2$ | 8.757 | 8.966 | 107.2 | 2e-16*** | 0.0711 | 7.12 | 122739 |
| $PM_{2.5}$ | 8.856 | 8.988 | 620.9 | 2e-16*** | | | |
| $NO_2$ | 8.593 | 8.909 | 456.1 | 2e-16*** | 0.0455 | 4.56 | 122014 |
| DEM | 8.924 | 8.998 | 240.8 | 2e-16*** | | | |
| $NO_2$ | 8.491 | 8.862 | 559.9 | 2e-16*** | 0.3140 | 31.40 | 122739 |
| SH | 8.889 | 8.996 | 5673.7 | 2e-16*** | | | |
| $NO_2$ | 8.502 | 8.867 | 618.9 | 2e-16*** | 0.0820 | 8.21 | 122739 |
| PREC | 8.960 | 8.999 | 788.7 | 2e-16*** | | | |
| $NO_2$ | 8.761 | 8.967 | 287.0 | 2e-16*** | 0.0695 | 6.96 | 122379 |
| NDVI | 8.904 | 8.997 | 598.1 | 2e-16*** | | | |
| $SO_2$ | 8.853 | 8.988 | 287.0 | 2e-16*** | 0.0831 | 8.33 | 122739 |
| $PM_{2.5}$ | 8.829 | 8.983 | 791.9 | 2e-16*** | | | |
| $SO_2$ | 8.879 | 8.992 | 413.6 | 2e-16*** | 0.0429 | 4.30 | 122014 |
| DEM | 8.853 | 8.994 | 175.8 | 2e-16*** | | | |
| $SO_2$ | 8.860 | 8.989 | 455.7 | 2e-16*** | 0.309 | 31.00 | 122739 |
| SH | 8.859 | 8.994 | 5522.0 | 2e-16*** | | | |
| $SO_2$ | 8.851 | 8.988 | 436.0 | 2e-16*** | 0.0706 | 7.07 | 122739 |
| PREC | 8.946 | 8.999 | 595.6 | 2e-16*** | | | |
| $SO_2$ | 8.831 | 8.985 | 390.6 | 2e-16*** | 0.0764 | 7.66 | 122379 |
| NDVI | 8.940 | 8.999 | 681.5 | 2e-16*** | | | |
| $PM_{2.5}$ | 8.876 | 8.991 | 933.5 | 2e-16*** | 0.0772 | 7.73 | 122014 |
| DEM | 8.940 | 8.999 | 204.4 | 2e-16*** | | | |
| $PM_{2.5}$ | 8.894 | 8.994 | 921.2 | 2e-16*** | 0.332 | 33.20 | 122739 |
| SH | 8.878 | 8.996 | 5462.2 | 2e-16*** | | | |
| $PM_{2.5}$ | 8.891 | 8.993 | 998.6 | 2e-16*** | 0.106 | 10.60 | 122739 |
| PREC | 8.948 | 8.999 | 647.8 | 2e-16*** | | | |
| $PM_{2.5}$ | 8.802 | 8.978 | 689.2 | 2e-16*** | 0.0957 | 9.58 | 122379 |
| NDVI | 8.884 | 8.996 | 483.3 | 2e-16*** | | | |
| DEM | 8.892 | 8.997 | 274.6 | 2e-16*** | 0.3010 | 30.10 | 122014 |
| SH | 8.871 | 8.995 | 5567.8 | 2e-16*** | | | |
| DEM | 8.884 | 8.996 | 194.3 | 2e-16*** | 0.0546 | 5.47 | 122014 |
| PREC | 8.909 | 8.997 | 586.4 | 2e-16*** | | | |
| DEM | 8.837 | 8.992 | 240.9 | 2e-16*** | 0.0669 | 6.70 | 122014 |
| NDVI | 8.865 | 8.995 | 774.2 | 2e-16*** | | | |
| SH | 8.883 | 8.996 | 4851.8 | 2e-16*** | 0.2930 | 29.30 | 122739 |
| PREC | 8.973 | 9.000 | 118.2 | 2e-16*** | | | |
| SH | 8.816 | 8.990 | 5988.0 | 2e-16*** | 0.3400 | 34.00 | 122379 |

| | | | | | | | |
|---|---|---|---|---|---|---|---|
| NDVI | 8.719 | 8.977 | 1109.0 | 2e-16*** | | | |
| PREC | 8.974 | 9.000 | 1132.0 | 2e-16*** | 0.1230 | 12.30 | 122379 |
| NDVI | 8.915 | 8.998 | 1270.0 | 2e-16*** | | | |

Note: DEM, Digital Elevation Model; SH, sunshine hours; PREC, precipitation; f. NDVI, normalized difference vegetation index; ***, significant at the 99.9% confidence level.

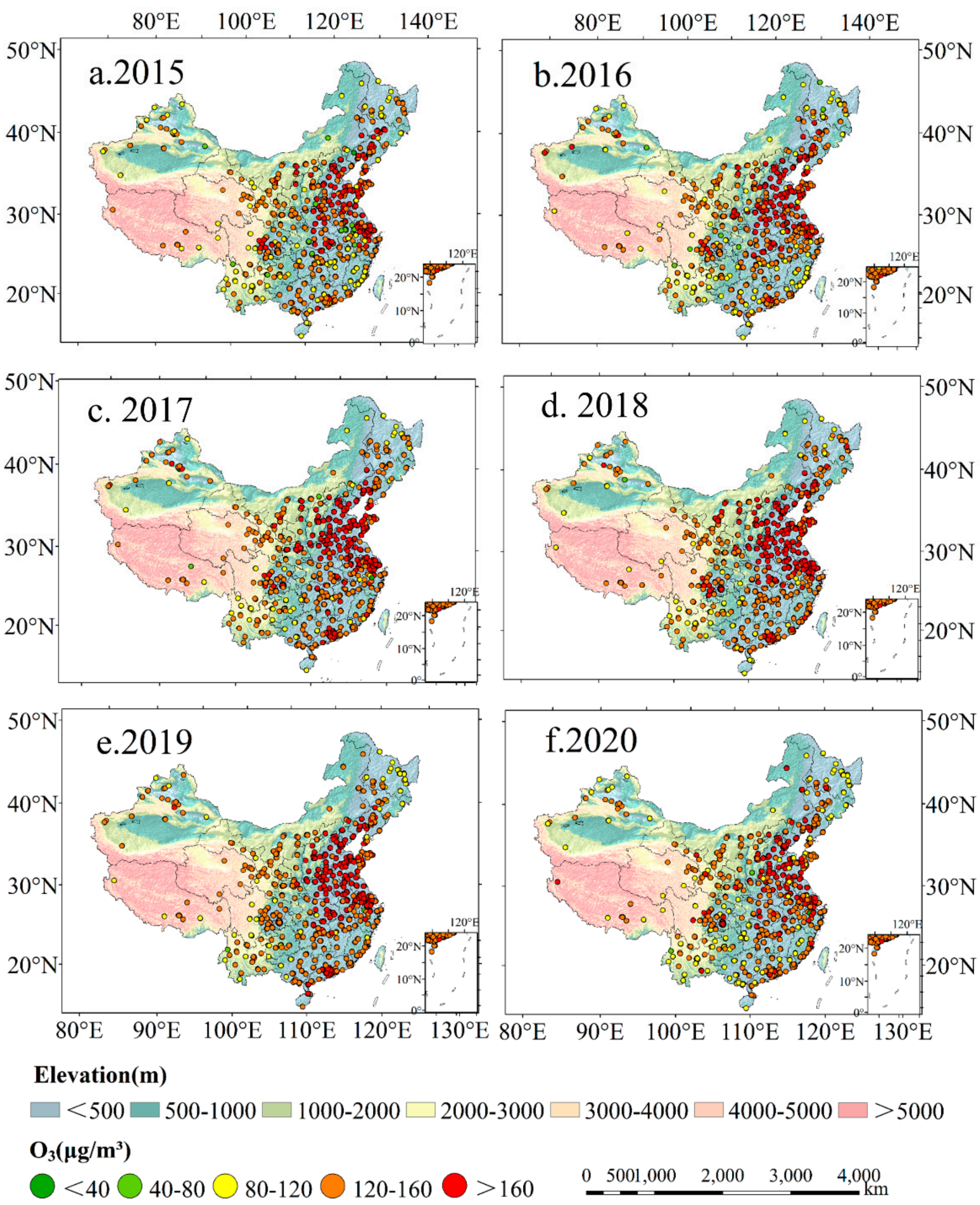


Figure S1 Distribution characteristics of the $O_3$ concentration (the 90th percentile of daily maximum 8-hour average) (90th MDA8) at various urban monitoring sites in mainland China from 2015 to 2020.

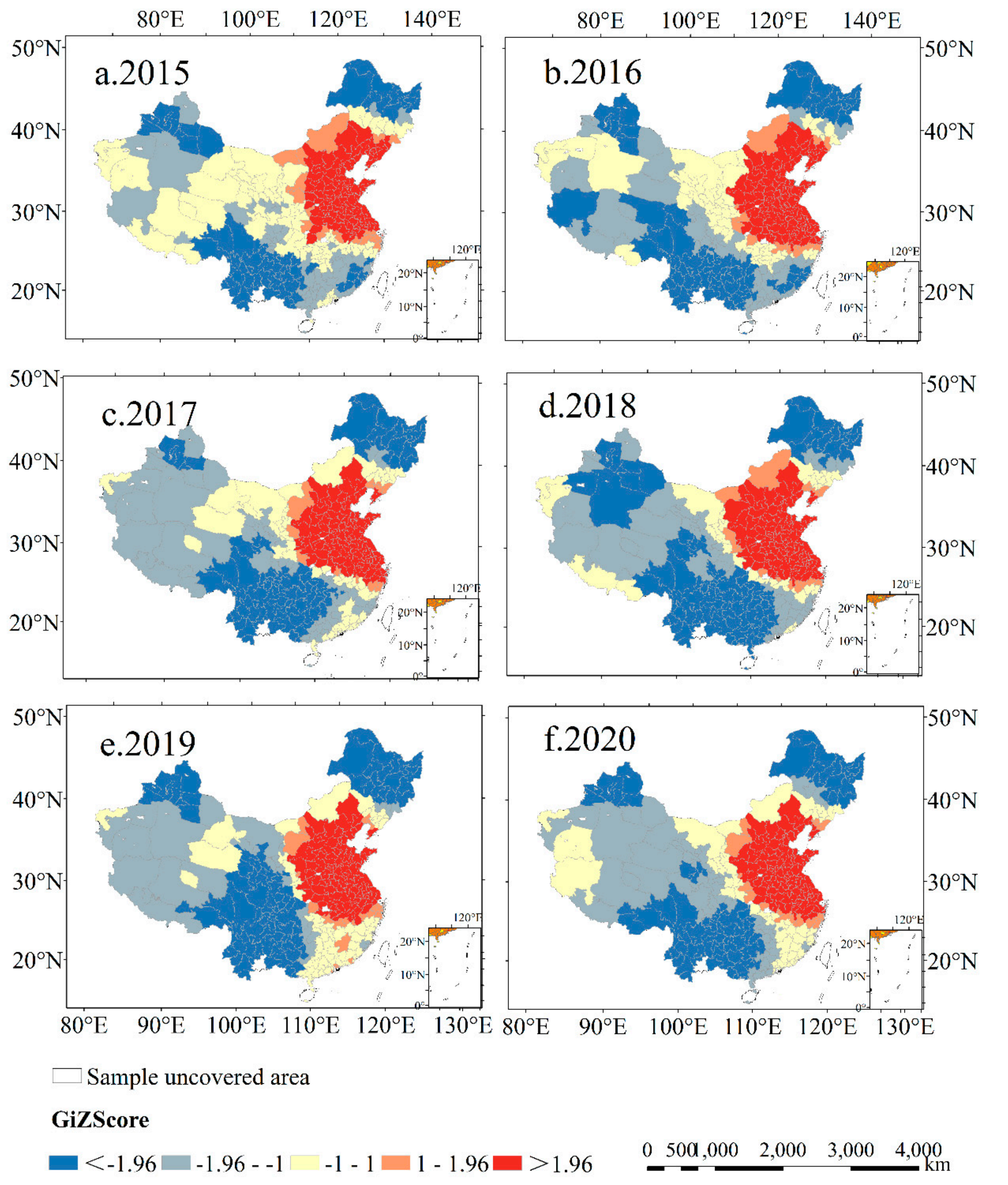


Figure S2 The hot point analysis of urban $O_3$ distribution in mainland China from 2015 to 2020.

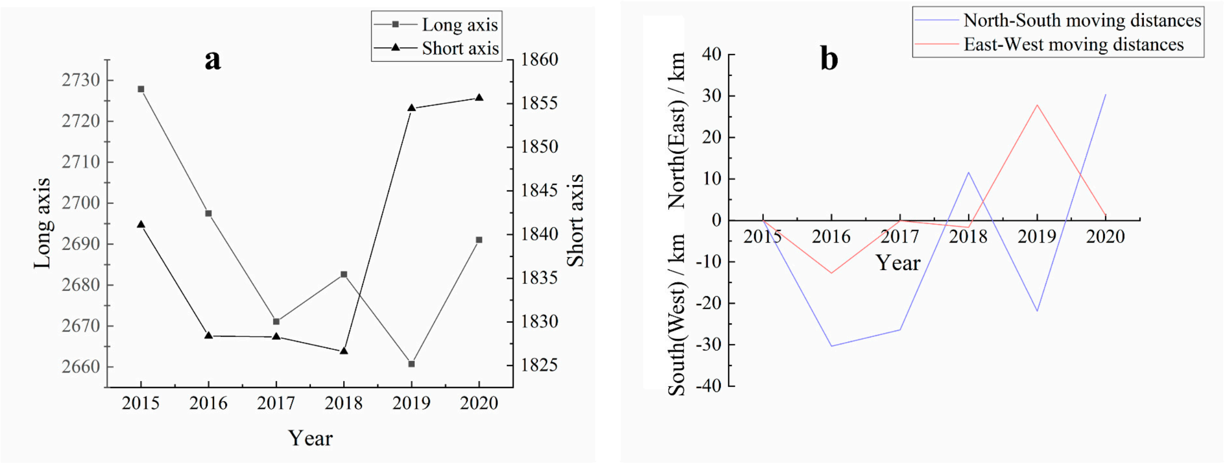


Figure S3 Long and short axes variation characteristics (a) and mean center movement characteristics (b) of standard deviation ellipse.

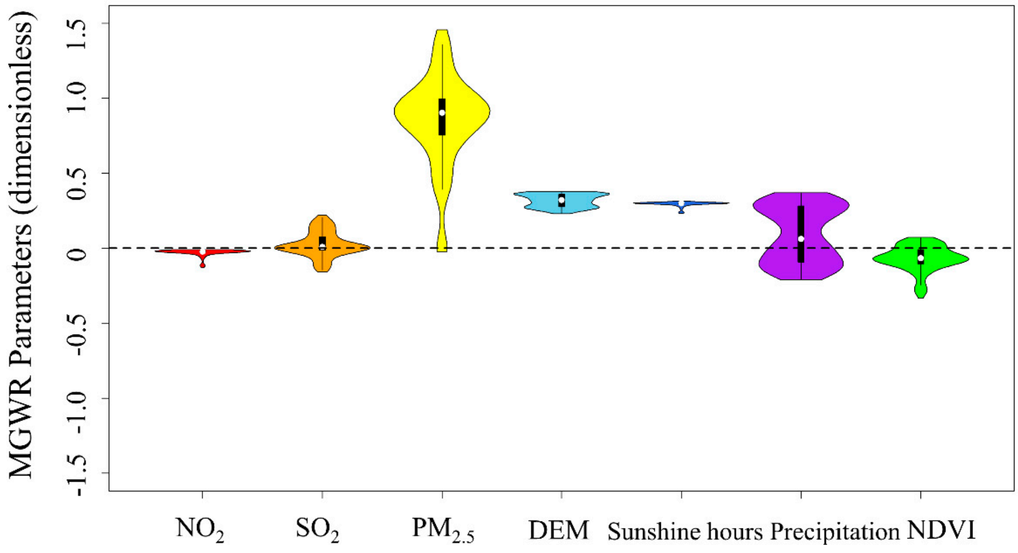


Figure S4 Vioplot of multi-scale geographic weighted regression coefficients

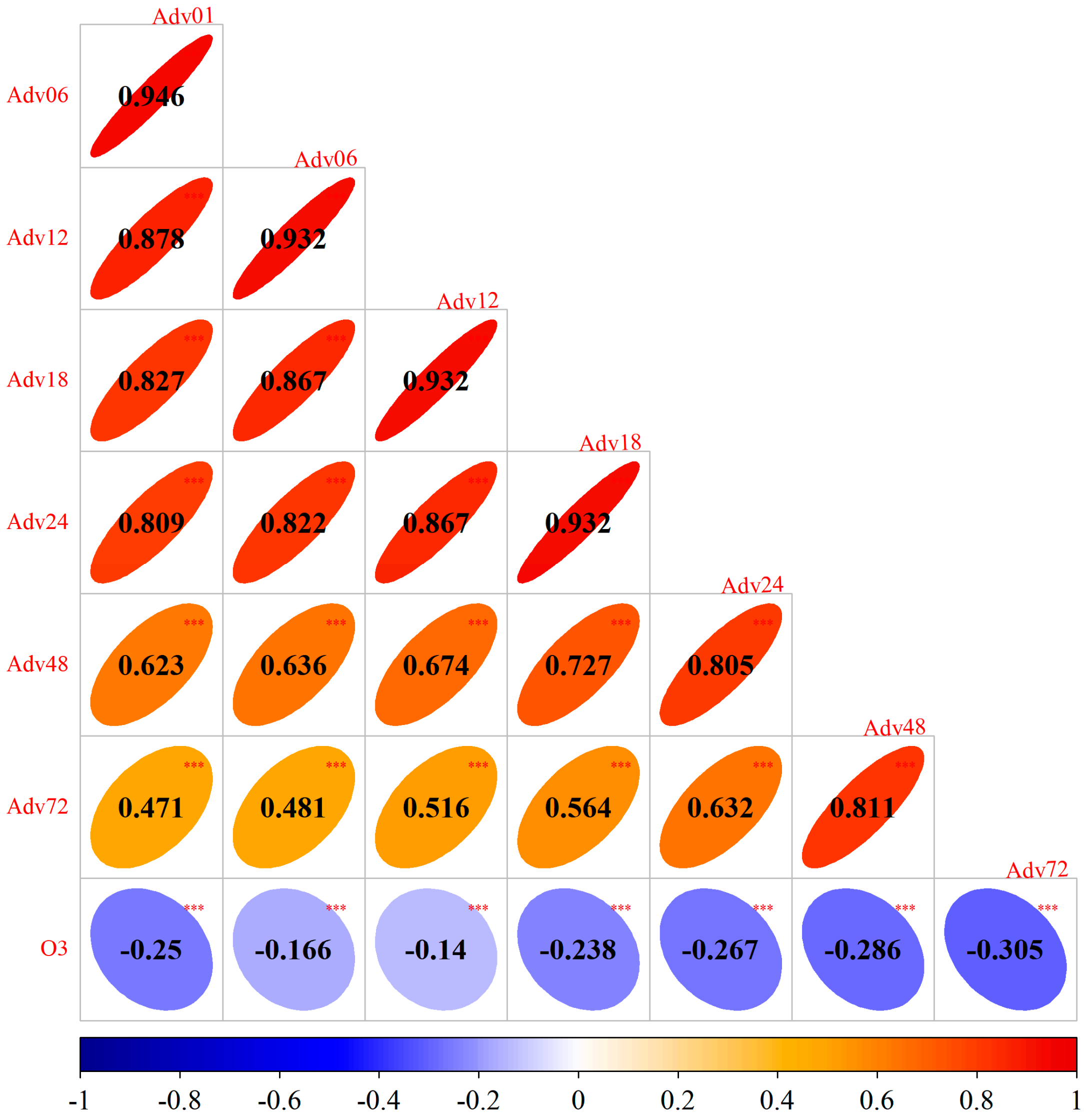


Figure S5 Correlation analysis of $O_3$ concentrations with $PM_{2.5}$ at 1, 6, 12, 18, 24, 48 and 72 hours in advance in the Yangtze River Delta region in 2018.(Note: Adv01, Adv06, Adv012, Adv018, Adv24, Adv48 and Adv72 represent urban $PM_{2.5}$ concentration at 1, 6, 12, 18, 24, 48 and 72 hours in advance, respectively.)

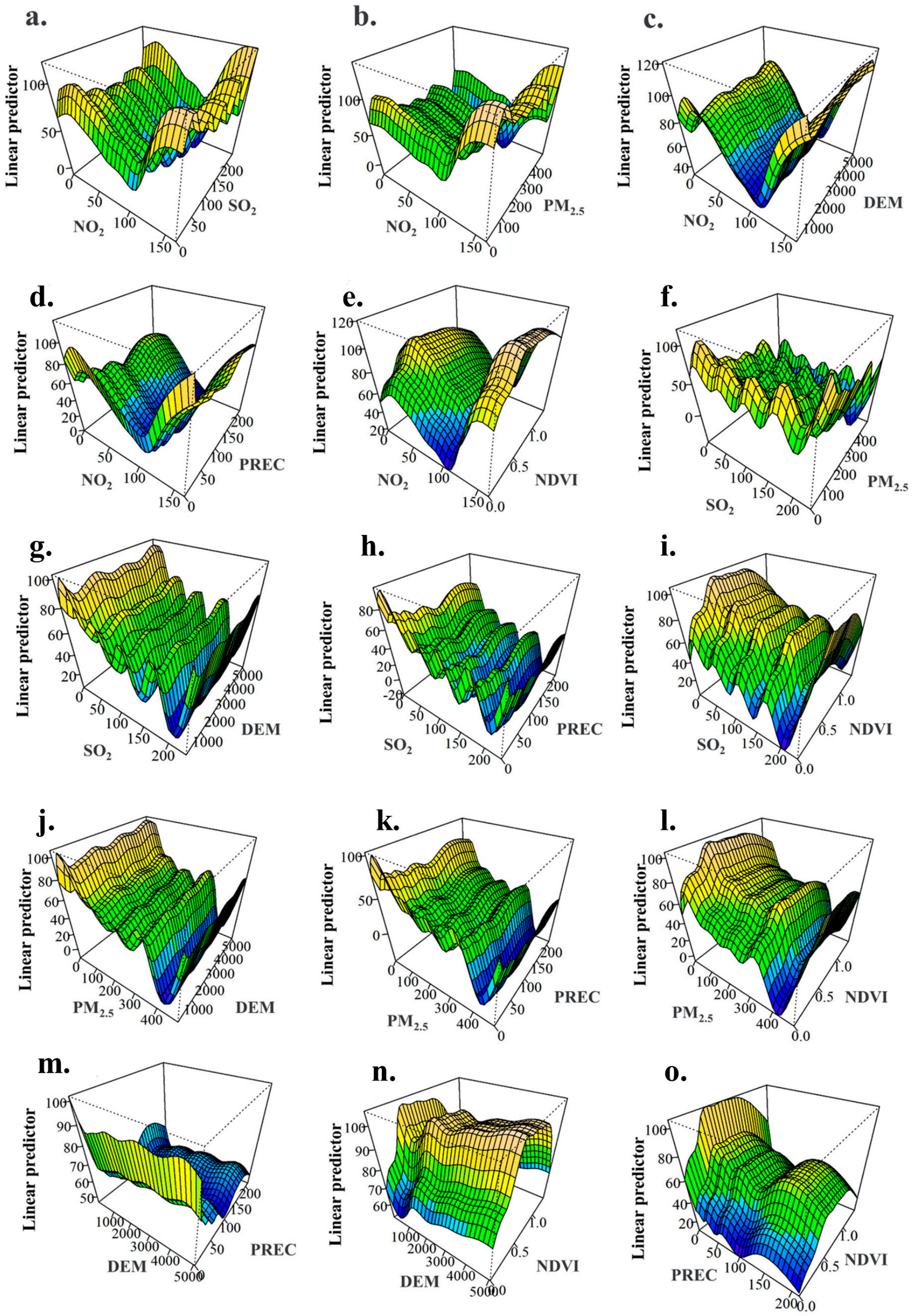


Figure S6. Interaction of different factors on the variation of urban $O_3$ concentration three-dimensional effect plots. a. $NO_2$ and $SO_2$; b. $NO_2$ and $PM_{2.5}$; c. $NO_2$ and Digital Elevation Model (DEM); d. $NO_2$ and precipitation (PREC); e. $NO_2$ and normalized difference vegetation index (NDVI); f. $SO_2$ and $PM_{2.5}$; g. $SO_2$ and DEM; h. $SO_2$ and PREC; i. $SO_2$ and NDVI; j. $PM_{2.5}$ and DEM; k. $PM_{2.5}$ and PREC; l. $PM_{2.5}$ and NDVI; m. DEM and PREC; n. DEM and NDVI; o. PREC and NDVI.

**References:**

Anand, V., Panicker, A.S. and Beig, G. (2020) Gaseous pollutants over different sites in a metropolitan region (Pune) over India. Sn Applied Sciences 2(4).

Andreev, V.V., Arshinov, M.Y., Belan, B.D., Davydov, D.K., Elansky, N.F., Zhamsueva, G.S., Zayakhanov, A.S., Ivlev, G.A., Kozlov, A.V., Kotel'nikov, S.N., Kuznetsova, I.N., Lapchenko, V.A., Lezina, E.A., Postylyakov, O.V., Savkin, D.E., Senik, I.A., Stepanov, E.V., Tolmachev, G.N., Fofonov, A.V., Chelibanov, I.V., Chelibanov, V.P. and Shirotov, V.V. (2020) Surface Ozone Concentration over Russian Territory in the First Half of 2020. Atmospheric and Oceanic Optics 33(6), 671-681.

Ezimand, K. and Kakroodi, A.A. (2019) Prediction and spatio - Temporal analysis of ozone concentration in a metropolitan area. Ecological Indicators 103, 589-598.

Kobza, J., Geremek, M. and Dul, L. (2021) Ozone Concentration Levels in Urban Environments-Upper Silesia Region Case Study. International Journal of Environmental Research and Public Health 18(4).

Krug, A., Fenner, D., Mucke, H.G. and Scherer, D. (2020) The contribution of air temperature and ozone to mortality rates during hot weather episodes in eight German cities during the years 2000 and 2017. Natural Hazards and Earth System Sciences 20(11), 3083-3097.

Sicard, P., Serra, R. and Rossello, P. (2016) Spatiotemporal trends in ground-level ozone concentrations and metrics in France over the time period 1999-2012. Environmental Research 149, 122-144.
